# Management of Ankle Charcot Neuroarthropathy: A Systematic Review

**DOI:** 10.3390/jcm10245923

**Published:** 2021-12-17

**Authors:** Ahmed ElSayed Galhoum, Vineet Trivedi, Mohamed Askar, Sergio Tejero, Mario Herrera-Pérez, Yousef AlRashidi, Victor Valderrabano

**Affiliations:** 1 George Eliot Hospital NHS Trust, Nuneaton CV114LG, UK; dm_askar@yahoo.com; 2 Ashford and St. Peter Hospital, NHS, London TW153AA, UK; rivedi_vineet@yahoo.com; 3 Foot Ankle Unit, University Hospital Virgen del Rocío, 41013 Seville, Spain; tejerogarciasergio@gmail.com; 4 University Hospital La Laguna, 38320 Canarias, Spain; herrera42@gmail.com; 5 Orthopaedic Department, College of Medicine, Taibah University, P.O. Box 30001, Almadinah Almunawwarah 41411, Saudi Arabia; yalrashidi@gmail.com; 6 Swiss Ortho Center, Schmerzklinik Basel, Swiss Medical Network, 4010 Basel, Switzerland; vvalderrabano@swissmedical.net

**Keywords:** Charcot neuroarthropathy, Charcot ankle, Charcot joint, Ilizarov, TCC

## Abstract

Background: Charcot neuroarthropathy is a non-infective, destructive process occurring in patients rendered insensate by peripheral neuropathy, which is caused mainly by diabetes. Repetitive trauma from standing and walking provides a neuro-traumatic stimulus that leads to dislocation, or peri-articular fracture, or both, within the ankle. This review concentrates on the management protocols regarding the ankle only. Methods: A Pubmed search for clinical trials performed to manage ankle Charcot neuroarthropathy and a systematic review of these articles were undertaken. Results: Twenty papers met the inclusion criteria: four of them describe non-surgical management, while the rest show different surgical management options of ankle Charcot neuroarthropathy. Conclusions: Surgical algorithms for the treatment of CN of the ankle are based almost entirely on level four. There is inconclusive evidence concerning the timing of treatment and the use of different fixation methods. Instability and ulceration are the main precursors for surgical interventions. Prospective series and randomized studies, albeit difficult to perform, are necessary to support and strengthen current practice.

## 1. Introduction

Charcot neuroarthropathy (CN) is a destructive, non-infective process affecting bones and joints that occurs in association with a peripheral neuropathy [1]. A peripheral neuropathy secondary to diabetes mellitus is the most common etiology of Charcot neuroarthropathy; however, peripheral neuropathy from leprosy, alcoholism, syringomyelia, rheumatoid arthritis, multiple sclerosis, and traumatic injury may also be associated with Charcot neuroarthropathy [1].

Recently, the American Diabetes Association estimated that nearly 7.8% of the population of the United States is affected by diabetes [2], and Charcot neuroarthropathy is thought to affect 8.5 per 1000 of the diabetic population per year [3].

The pathogenesis of Charcot arthropathy may be explained by neurotraumatic and neurovascular theories. Both mechanisms likely contribute to the syndrome [4]. With respect to the neurotraumatic theory, Gupta [5] noted that the common form of pathogenesis includes repeated injuries caused by minor trauma or by isolated major trauma to neuropathic joints. Many of the authors think that Charcot arthropathy may be triggered in diabetic patients by some type of joint trauma, and this is supported by the lack of protective sensations, which is a predisposing factor of the disease [5,6].

The neurovascular theory is based on the presence of vasomotor neuropathy in individuals with sensory neuropathy and intact blood flow. Vasomotor neuropathy produces arterio-venous shunting, leading to bone resorption [7] and mechanical weakening. The weakened bone may fracture and deform with continued weight bearing. Increased venous pressure associated with autonomic neuropathy also may increase capillary pressure and promote leg edema [8].

The diagnosis of Charcot arthropathy is primarily reliant on clinical presentation. A thorough patient history is essential to any assessment; however, a neuropathic patient’s history can be unintentionally misleading. Consequently, the clinician should make sure the patient’s answers to the questions are reliable.

Attention must especially be paid to the answers if the patient has an evident history of trauma, a history of neuropathy, recent swelling, and redness in the limb. Strangely, the history may include pain sensations in an insensate limb that were not caused by an obvious traumatic event. In a study of 55 patients with Charcot arthropathy, more than 75% complained of pain in the foot and ankle upon presentation, even though all subjects had a clinical loss of protective sensation to the 10 g Semmes-Weinstein mono-filament wire [9].

Repetitive trauma to the foot and ankle may be entirely absent from a verbal history, even though clinical symptoms prove it occurred. Only 22% of patients were able to recall some specific traumatic event prior to the onset of Charcot arthropathy. The loss of protective sensation makes the patient unaware of some traumatic events [10].

Infection should be ruled out because the presentations of Charcot neuroarthropathy and acute soft tissue infection are similar. An investigation of any previous history of infection or ulcers to exclude a recurring acute or chronic infection is important [9].

Most infections in a diabetic foot or ankle involve a direct source of infection through a skin compromise, usually caused by neuropathic ulcers [11,12].

Non-weight-bearing radiographs may not show any subtle instability compared to the weight-bearing positions. It is recommended that all radiographic examinations of feet and ankles be obtained in a weight-bearing position, if possible. On the other hand, MRI examinations are increasingly being used and recommended for diagnosing Charcot arthropathy, especially at the earliest stage [13].

There is no consensus or guideline for management of ankle CN, or to define the proper stage for surgical intervention. Off-loading orthoses, such as total contact casts, braces, or Charcot restraint orthotic walker (CROW), are widely thought to be the initial treatment methods [14]. Medications, such as pamindronate, are among the suggested therapies [15,16]. The role of and time for surgical intervention are not clear and intervention has unpredictable results. However, many salvage procedures have been described, including open reduction internal fixation (ORIF), with variable techniques and implants [17,18,19], and an external-fixation strategy [20,21,22,23].

The aim of this study is to evaluate the current modalities for treatment of ankle CN, with analysis of each protocol and result, and to assess the surgical options in correlation to functional and clinical outcomes, limitations, and complications, which may help in future decision-making.

## 2. Materials and Methods

PubMed searches were done with the following keywords: “Management of, treatment of, Charcot ankle, Charcot ankle neuro-arthropathy and ankle neuroarthropathy”

### 2.1. Points of Comparison 

In this study, the points of comparison were patient demographics (age and sex), follow-up periods, type of treatment, follow-up data, and results.

### 2.2. Inclusion Criteria 

The inclusion criteria for the selected articles were:

1. Articles from 1995–2020;
2. English literature only;
3. Human studies;
4. Clinical trials;
5. Orthopedic journals only.

### 2.3. Exclusion Criteria 

The exclusion criteria were:

1. In vitro studies;
2. Duplicated articles by the same authors unless they had longer follow-up studies;
3. Technical notes.

Searches using the keywords in different combinations yielded 943 articles. By checking the titles, these were narrowed down to 577 research articles. The application of the inclusion and exclusion criteria resulted in 20 papers about Charcot arthropathy of the ankle joint. We classified them according to different methods of management:

- Non-surgical management of Charcot arthropathy (4 papers);
- Surgical management of Charcot arthropathy (16 papers);

According to the level of evidence, we found:

1. Level of evidence I: 1 paper;
2. Level of evidence III: 3 papers;
3. Level of evidence IV: 16 papers.

## 3. Results

The total number of patients included in these studies was 405. Their demographic description and distribution appear in Table 1 and Table 2. The studies were allocated in two groups: non-surgical interventions including medications and orthotic methods, and surgical interventions.

### 3.1. Group 1: Non-Surgical Management of Charcot Arthropathy

Four papers are included in this group. One of them is a prospective study with level IV evidence, one is double-blinded randomized controlled trial with level I evidence, and two are retrospective studies with level II evidence III. The details of these papers are provided in Table 3 showing the differences between evaluation methods, numbers of patients involved, management methods, follow-up data, follow-up periods, and end results.

### 3.2. Group 2: Surgical Management of Charcot Arthropathy

Sixteen papers are included in this group. Ten of them are prospective studies with level IV evidence, one is a level III cohort study, and five are level IV retrospective case series. Details of these articles are depicted in Table 4, showing the difference between evaluation methods, numbers of patients involved, surgical techniques used, follow-up data, follow-up periods, and results.

## 4. Discussion

The management of patients with foot and ankle diabetic neuroarthropathy is challenging. Educating patients about prevention, early recognition of arthropathy, and prompt institution of protective treatment are clearly crucial factors that determine the outcome of this problem. The mainstay of treatment for ankle Charcot neuroarthropathy is prolonged immobilization in the form of a plaster cast, or brace, or the use of antiresorptive medication during the acute stage. However, some patients already have disabling deformity or severe instability at the time of presentation, for which conservative treatment alone is destined to fail. For such patients, reconstruction of the foot and ankle is a valuable technique [22].

In this review, 405 patients underwent different treatment modalities, of which 110 experienced non-surgical treatment with variable conservative modalities, while the remaining 295 underwent surgical treatment with different fixation modalities and operative techniques.

Regarding non-surgical treatment, Jude et al. [15] and Anderson et al. [16] studied the effect of bisphosphonate on 62 patients regarding its role in improving clinical signs during the acute stage. Thirty-four patients received bisphosphonate while the other twenty-eight (the control group) were given a placebo. All patients who received bisphosphonate showed a 100% decrease in the clinical signs and symptoms of Charcot arthropathy compared to the placebo group.

The exact mechanisms by which bisphosphonate inhibits bone resorption are unknown. It is known that pamidronate is taken up by bone, is bound to the hydroxyapatite crystal of the bone matrix, and then acts to prevent osteoclast precursors from attaching to bone. Pamidronate also directly inhibits mature, already active, osteoclasts, and promotes osteoclast apoptosis. Finally, pamidronate decreases osteoblast-mediated osteoclast activation. Although pamidronate inhibits osteoclasts via several mechanisms, it has not been shown to impair mineralization [34,35,36].

Inflammation regression in the form of temperature drop was clinically recorded in the members of the Charcot ankle arthropathy group who received pamidronate. A decrease of alkaline phosphatase was also noted in the same group. The single infusion of 60–90 mg intravenously over 4–24 h was the selected regimen, as reported by Jude et al. [15] and Anderson et al. [16] in their studies.

There was no complication in the bisphosphonate treatment reported by Jude et al. [15], while Anderson et al. [16] reported a post-administration systemic fever of 1–3 °F, which subsided within 24 h after slow infusion. This fever occurred in 9.67% of the study population (6 out of 62 patients). Transient nausea and gastrointestinal upset were also observed in 8.06% of their patients, who experienced them for a short time. There were no major side effects related to pamidronate in any of the study patients. Despite the effect of pamidronate on the acute process of Charcot arthropathy, there are several concerns regarding Jude et al.’s and Anderson et al.’s methodology. First, the pamidronate group and the placebo group were in different institutions and were not studied concurrently. Second, they had 62 patients in their review, which is a small number. Consequently, larger trials would be necessary to show sufficient power in the results. Lastly, the sensor devices were not calibrated because the tools were actually different at each site, and some variability may have existed.

De Souza et al. [27] evaluated weight-bearing total-contact casts (TCCs) and orthosis as other modalities of conservative treatment. The evaluation included 27 patients with Charcot arthropathy (7 patients with bilateral ankle involvement). The 34 feet involved showed no deleterious effect from weight bearing in 100% of cases, so TCC is considered a safe immobilization technique in Eichenholtz stage-1 Charcot arthropathy of the ankle. Ulcer development was noted in 10 out of 34 feet (29.41%). Yet, none of the patients complained during the TCC application period (14 weeks). They all developed after the limb had been placed in an orthosis (at an average of 6.5 weeks). Although we favor the idea that weight bearing causes no harm, some limitations were expressed by de Souza et al. [27]: First, it was difficult to distinguish the end of stage I and the beginning of stage II, so the period of treatment with weight-bearing TCC was extended to include the earlier part of stage II. Second, noncompliance showed by some patients led to irregular attendance on scheduled clinic dates.

An alternative to TCC is a prefabricated pneumatic walking brace. Verity et al. [14] evaluated this modality of conservative treatment on 21 patients (4 bilateral). The majority of patients (84%) were satisfied with the brace, while 8% found it moderately helpful, 4% not helpful at all, and 4% reported it aggravated the condition.

The authors reported some complications as 7 out of 25 feet (28%) developed ulcers, while 21 feet (84%) developed different types of pain (musculoskeletal pain and pain from the direct pressure of the brace). The pneumatic walker brace may be more economical over the course of treatment compared with TCC. However, a formal comparative economic analysis was not conducted. Moreover, TCC showed more complications compared to pneumatic walker braces regarding ulcer development. The authors also admitted in the study to the presence of some limitations, as the brace may be relatively contraindicated in patients with hypermobile instability. It is important that the procedure properly realigns the foot and ankle to prevent bony protuberances that could lead to ulcerations and subsequent abscesses or osteomyelitis.

Out of 405 patients, 295 underwent different surgical fixation techniques in this review. Pawar et al. [19], Pinzur et al., 1997 [24], Caravaggi et al., 2006 [18], Pinzur et al., 2005 [25], Caravaggi et al., 2012 [28], Dalla Paola et al. [26], Siebachmeyer et al. [29], DeVries et al. [31], and Shah et al. [32] evaluated 181 patients (2 patients with bilateral ankle involvement) for the results of using an intramedullary nail as a stabilization method and they achieved a high fusion rate.

The access for the intramedullary nail (Ankle Arthrodesis Nail, Orthofix Inc., Bussolengo, Italy) was created along the Kirschner guidewire passed to the proximal tibia, and the intramedullary canal was reamed. A nail with a diameter 1 mm smaller than the final reamer and a length determined by a radiolucent ruler was inserted, with the average length being 140, 160, or 180 mm. The nail was placed with the bow directed posteriorly to augment the posterior displacement of the foot on the leg. Percutaneous distal locking of the nail was achieved using the insertion/aiming guide. Proximal locking was accomplished using a freehand technique monitored with image intensification.

Solid fusion was achieved in 152 out of 183 feet (83.06%). Stiff fibrous union was obtained in 13 feet (7.1%), and only 16 feet (8.74%) underwent below-knee amputation. One patient showed non-union (0.54%), and one patient died. The complications noted by the authors in our review when using intramedullary nail fixation are variable. Infection is the most common complication reported in their patients at the rate of 45.85%, presented in the form of superficial wound infection, or the loosening and breakage of proximal or distal screws. Pawar et al. [19] overcame the infection problem by using antibiotic-coated intramedullary nails and all their patients did well and showed 100% complete healing. Despite these good results, the limitation of their study is the small patient number (five only). So, future studies with larger numbers are required.

The other complications of intramedullary nails are ulcer development in 15 out of 181 patients (8.27%), post-operative hematoma in 1 patient (0.55%), and 16 patients (8.74%) underwent knee amputation.

The results shown here suggest that treatment of ankle CN with arthrodesis using retrograde nailing is a safe and effective option when gross ankle instability is evident.

Another internal fixation method is ankle arthrodesis using crossed screws. Ayoub et al. [17] demonstrated the results of an attempt to salvage the limbs of 17 patients using cannulated screws to obtain tibiotalar fusion. Solid fusion was achieved in nine patients (53%), and higher fusion rates were achieved with three screws. A stiff fibrous union was obtained in five patients (29.4%). Only three patients (18%) developed unstable pseudoarthroses, which led to below-knee amputation. The complications encountered in their cohort were superficial wound infections in four patients (23.5%), and avascular necrosis of talus, as well as hind foot ulceration, in three patients (17.6%).

The blade plate ankle arthrodesis technique was evaluated by Cinar et al. [30] and Myerson et al. [33] in an attempt to salvage 30 ankles through tibiotalar fusion. All patients achieved limb salvage (100%), either by solid fusion (in 27 patients: 90%), or stiff fibrous union (in three patients: 10%). Infection was the most common complication seen in 18 out of 30 patients (60%). Another complication was a stress fracture at the proximal end of the plate (6.66%). The limitation of these studies is the small population number in each of them, so future studies with larger numbers are still required. However, these results are satisfactory and represent a reliable treatment approach.

Nevertheless, open correction with internal fixation for Charcot osteoarthropathy is associated with appreciable rates of complications and failures because of infection, bone softening, resorption, fragmentation, and breakage of implants. Complex reconstructive procedures with arthrodesis are more frequently reserved for realignment and stabilization of severely deformed feet and ankles in an effort to avoid amputation.

The choice of internal or external fixation depends on the quality of bone. Generally, in Charcot disease, the bone stock is poor and external fixation provides better compression with fewer fixation failures and soft tissue complications. Because of its ability to correct multiplanar deformities in osteopenic bone, even in the presence of open wounds, the circular (Ilizarov) external fixator offers an excellent option for Charcot foot and ankle reconstructions. We reviewed six studies comprising 67 patients who underwent surgical reconstruction using external fixators. Yousry et al. [22], El-Gafary et al. [20], Fabrin et al. [21], Zarutsky et al. [23], De Vries et al. [31], and Shah et al. [32] reported solid fusion and anatomical reduction in 48 patients (71.64%), fibrous union in 10 patients (14.92%), non-union in 4 patients (5.97%), and below-knee amputation in 6 patients (8.95%).

Using a ring external fixator to correct and stabilize foot and ankle deformities is effective as it facilitates correction of deformities and avoids the complications encountered with internal fixation. It also allows early weight bearing, care of soft tissue, prevention of skin ulceration, and avoidance of amputation. However, it should be recognized that external fixation is not without disadvantages since it involves a lengthy treatment with a mean follow-up period of 24 months, which, as noted in the results, is commonly associated (64.17%) with pin-tract infection. An external ring fixation also requires surgical expertise and dedicated instrumentation. Nevertheless, these problems may be outweighed by the advantages of the technique.

## 5. Conclusions

Early recognition and prevention of collapse are still the best options for the management of patients with diabetic Charcot arthropathy of the ankle. In patients with diabetes and lower extremity neuropathy, any minor injury requires careful observation because of the tendency of the limb to proceed to a Charcot process, so appropriate education, improved clinical evaluation, and early intervention are required to control the disease.

Once collapse is present, the use of an off-loading TCC and anti-resorptive medication is recommended in the acute stage. In the following stages, procedures for the salvage of the CN ankle with ORIF and external fixation are valuable options that should be tried before deciding on amputation, unless there is severe vascular impairment or unmanageable infection.

## Figures and Tables

**Table 1:** Overall descriptive analysis.

Total Number of Patients	405
Mean age	55.01
Male:Female	1.19:1
Mean follow-up time in months	22.07

**Table 2:** Detailed patient distribution in each study according to age, sex, and follow-up periods.

Article		Number	Mean Age (Years)	Sex		Months of Follow-Up
				M	F	
1	El-Gafary et al. [20]	20	30	11	9	20
2	Ayoub et al. [17]	17	61.6	7	10	26
3	Pinzur et al., 1997 [24]	9	52	4	5	32
4	Pinzur et al., 2005 [25]	20	56.3	9	11	31
5	Caravaggi et al., 2006 [18]	14	58	13	1	18 ± 4
6	Jude et al. [15]	39	56	26	13	12
7	Pawar et al. [19]	5	59	4	1	12–24
8	Dalla Paola et al. [26]	18	65.3	13	5	14 ± 10.1
9	Fabrin et al. [21]	11	61	4	7	48
10	De Souza et al. [27]	27	-	6	21	5.5
11	Anderson et al. [16]	23	-	13	10	75
12	Caravaggi et al. [28]	45	56	27	18	5 ± 3
13	Yousry et al. [22]	12	-	4	8	19.3
14	Verity et al. [14]	21	52	10	11	33
15	Siebachmeyer et al. [29]	20	62.6	12	8	26
16	Cinar et al. [30]	4	63	2	2	24
17	DeVries et al. [31]	52	55.5	30	22	24 ± 19.43
18	Zarutsky et al. [23]	11	57.3	7	4	27
19	Shah et al. [32]	11	56	6	5	4
20	Myerson et al. [33]	26	47.84	13	13	48
Total		405		221	184	441.55 ± 36.53
Average			55.84	1.2:1		22.07

**Table 3:** Group 1 studies with non-surgical intervention.

Study	Design	Pt. no.	Disease Stage	Treatment Applied	F/U Period	Results
**Jude et al. [15] (2001)**	Double-blinded RCT/level I	*n* = 39 Study = 21 Pl. = 18	I	Patients received 90 mg of pamidronate over 4–24 h as a single infusion dose or placebo (saline)	12 m	An improvement in symptoms was seen in the active group compared to the placebo group; reduction in bone turnover was greater in the active than in the control group
**Anderson et al. [16] (2004)**	Retrospective study/level III	*n* = 23 Study = 13 Control = 10	I	13 study patients administered pamidronate were compared with 10 control patients who were treated with traditional immobilization methods	3 wks	After pamidronate infusion, limb temperature decreased 7.4 in 2 weeks; The alkaline phosphatase also decreased an average of 53% 2 weeks after infusion; the control group showed no significance reduction of limb temperature or alkaline phosphatase
**Verity et al. [14] (2007)**	Retrospective study/level III	*n* = 21	III	Prefabricated pneumatic removable walker brace fitted with a custom orthotic insole	33 m	Patients’ subjective impressions of removable walker brace: Greatly helpful: 84% Moderately helpful: 8% Minimally helpful: 0% Not helpful at all: 4% Aggravated condition: 4%
**de Souza et al. [27] (2008)**	Prospective study/level IV	*n* = 27	I/II	Immobilization in a weight-bearing total-contact cast	5.5 m	No deleterious effect from weight bearing.

m = month, wks = weeks.

**Table 4:** Group 2 studies with surgical interventions.

Article	Study Design/Level of Evidence	Number of Patients	Stage Of Disease	Surgical Technique	F/U Period	Results
**(1) Caravaggi et al. [28] (2012)**	Cohort/level III	45	Unspecified	Tibiocalcaneal arthrodesis using retrograde intramedullary nail fixation	5 ± 2.85 y	4 patients (8.88%): below-knee amputation; 2 patients (4.44%): fibrous union and required pneumatic casts for ambulation 39 patients (86.67%): solid union and returned to independent ambulation wearing custom-made shoes with molded insoles
**(2) Pawar [19] (2013)**	Prospective study/level IV	5	Stage I/III	Retrograde antibiotic-coated locked intramedullary nail	12–24 m	All achieved infection control and bony union
**(3) Fabrin [21] (** **2007)**	Prospective study/level IV	11	Unspecified	Arthrodesis with external fixation	48 m	7 cases of tibiotalar arthrodesis were performed: 5 resulted in bony union, 2 resulted in fibrous union; 5 cases of tibiocalcaneal arthrodesis were performed: 1 resulted in bony union, 2 resulted in stable fibrous union, 1 resulted in unstable fibrous union, 1 resulted in amputation
**(4) Ayoub [17] (2008)**	Prospective study/level IV	17	Stage II/III	Tibiotalar arthrodesis (crossed screw technique)	26 m	Success rate: 82.4% 9 patients: bony union 5 patients: stiff fibrous union 3 patients: below-knee amputation
**(5) El-Gafary et al. [20] (2009)**	Prospective study/level IV	20	Stage II	Surgical arthrodesis by illizarove frame	20 m	100% success; all patients show solid union and correction of deformities
**(6) Pinzur et al. [25] (2005)**	Prospective study/level IV	9	Unspecified	Arthrodesis with retrograde intramedullary nailing	32 m	100% success; 9 patients show union fusion
**(7) Pinzur et al. [24] (1997)**	Prosepective study/level IV	20	Unspecified	Retrograde locked intramedullary nail	12–31 m	19 patients achieved bony fusion 1 patient:amputation 1 patient: died
**(8) Caravaggi et al. [18] (2006)**	Prospective study/level IV	14	Stage II	Intramedullary compressive nail fixation	18 ± 4 m	Success rate 92.2% 10 patients achieved solid arthrodesis, returning to walking with protective shoes 3 patients developed fibrous union that allowed walking with a brace 1 patient: below-knee amputation
**(9) Dalla Paola et al. [26] (2007)**	Prospective study/level IV	18	Stage IV	Panarthrodesis of ankle using intramedullary retrograde trans-calcaneal nailing	14 ± 10.1 m	100% limb salvage because of controled patient selection 14 patients: complete bony union of ankle arthrodesis 4 patients: fibrous union
**(10) Yousry et al. [22] (2010)**	Prospective study/level IV	12	Stage II/III	Tibiocalcaneal and tibitalar fusion using an illiazrove frame	19.3 m	Success rate 75% Fusion was confirmed in 9 patients (75%) 2 patients had pseudoarthrosis 1 patient had unstable pseudoarthrosis
**(11) Siebachmeyer et al. [29] (2015)**	prospective study/level IV	20	Unspecified	Retrograde intramedullary nail	26 m	100% salvage 19 feet showed bony fusion 1 foot showed stable fibrous union 1 foot showed nonunion
**(12) Zarutsky et al. [23] (2005)**	Retrospective analysis/level III	11	Unspecified	Circular wire External fixator	27 m	Bony union: 7 Fibrous union: 3 Amputation: 1
**(13) Shah et al. [32] (2011)**	Retrospective analysis/level III	11	Stage II/III	6 patients with external fixator 5 patients with retrograde intramedullary nail	4 m	Regarding IMN, all 5 patients achieved bony union (100%) Regarding external fixator, 6 patients: 1 patient: bony union 4 patients: nonunion 1 patient: amputation
**(14) Myerson et al. [33] (2000)**	Retrospective case series/level IV	26	Unspecified	Tibicalcaneal arthrodesis using a condylar blade plate	48 m	All achieved limb salvage 24 patients: bony union 2 patients: fibrous union
**(15) Cinar et al. [30] (2010)**	Retrospective case series/level IV	4	Unspecified	Tibicalcanel arthrodesis using posterior blade plate	24 m	All achieved limb salvage 3 patients: bony union 1 patient: fibrous union
**(16) DeVries et al. [31] (2012)**	Retrospective case series/level IV	52	Various stages	45 patients using retrograde intramedullary nail 7 patients using external fixator	24 m	Regarding intramedullary nail: 32 patients: stable bony union 3 patients: fibrous union 10 patients: amputation Regarding external fixator: 5 patients: bony union 2 patients: amputation

Y = year, IMN = intramedullary nail, m = month.

## Data Availability

Available on Public domain as cited in references, and upon request to corresponding author.

## References

[1] Brodsky J. (1992). Management of Charcot joints of the foot and ankle in diabetes. Semin. Arthroplast..

[2] Shaw J., Sicree R., Zimmet P. (2010). Global estimates of the prevalence of diabetes for 2010 and 2030. Diabetes Res. Clin. Pract..

[3] Lavery L.A., Armstrong D.G., Wunderlich R.P., Tredwell J., Boulton A.J. (2003). Diabetic Foot Syndrome: Evaluating the prevalence and incidence of foot pathology in Mexican Americans and non-Hispanic whites from a diabetes disease management cohort. Diabetes Care.

[4] Johnson J. (1967). Neuropathic fractures and joint injuries. J. Bone Jt. Surg. Am. Vol..

[5] Gupta R. (1993). A short history of neuropathic arthropathy. Clin. Orthop. Relat. Res..

[6] Simón-Pérez E., Simón-Pérez C., Alonso-Peña D., Pontón-Cortina A., Chicharro-Luna E., Martínez-Nova A., Navarro-Flores E. (2020). Stiffness degree of ankle range of motion in diabetic patients with atypical amputation. Rev. Da Assoc. Médica Bras..

[7] Edelman S.V., Kosofsky E.M., Paul R.A., Kozak G.P. (1987). Neuro-osteoarthropathy (Charcot’s joint) in diabetes mellitus following revascularization surgery: Three case reports and a review of the literature. Arch. Intern. Med..

[8] Edmonds M.E., Roberts V.C., Watkins P.J. (1982). Blood flow in the diabetic neuropathic foot. Diabetologia.

[9] Armstrong D.G., Todd W., Lavery L., Harkless L., Bushman T. (1997). The natural history of acute Charcot’s arthropathy in a diabetic foot specialty clinic. J. Am. Podiatr. Med. Assoc..

[10] Chantelau E., Richter A., Schmidt-Grigoriadis P., Scherbaum W.A. (2006). The Diabetic Charcot Foot: MRI Discloses Bone Stress Injury as Trigger Mechanism of Neuroarthropathy. Exp. Clin. Endocrinol. Diabetes.

[11] Sanders L. (1994). Diabetes mellitus. Prevention of amputation. J. Am. Podiatr. Med. Assoc..

[12] Singh N., Armstrong D.G., Lipsky B.A. (2005). Preventing Foot Ulcers in Patients with Diabetes. JAMA.

[13] Chantelau E., Poll L.W. (2006). Evaluation of the Diabetic Charcot Foot by MR Imaging or Plain Radiography—An Observational Study. Exp. Clin. Endocrinol. Diabetes.

[14] Verity S., Sochocki M., Embil J.M., Trepman E. (2008). Treatment of Charcot foot and ankle with a prefabricated removable walker brace and custom insole. Foot Ankle Surg..

[15] Jude E.B., Selby P., Burgess J., Lilleystone P., Mawer E.B., Page S.R., Donohoe M., Foster A.V.M., Edmonds M.E., Boulton A.J.M. (2001). Bisphosphonates in the treatment of Charcot neuroarthropathy: A double-blind randomised controlled trial. Diabetologia.

[16] Anderson J.J., Woelffer K.E., Holtzman J.J., Jacobs A.M. (2004). Bisphosphonates for the treatment of Charcot neuroarthropathy. J. Foot Ankle Surg..

[17] Ayoub M.A. (2008). Ankle fractures in diabetic neuropathic arthropathy. J. Bone Jt. Surg. Br. Vol..

[18] Caravaggi C., Cimmino M., Caruso S., Noce S.D. (2006). Intramedullary Compressive Nail Fixation for the Treatment of Severe Charcot Deformity of the Ankle and Rear Foot. J. Foot Ankle Surg..

[19] Pawar A., Dikmen G., Fragomen A., Rozbruch S.R. (2013). Antibiotic-Coated Nail for Fusion of Infected Charcot Ankles. Foot Ankle Int..

[20] El-Gafary K.A.M., Mostafa K.M., Al-Adly W.Y. (2009). The management of Charcot joint disease affecting the ankle and foot by arthrodesis controlled by an Ilizarov frame. J. Bone Jt. Surg. Br. Vol..

[21] Fabrin J., Larsen K., Holstein P.E. (2007). Arthrodesis with External Fixation in the Unstable or Misaligned Charcot Ankle in Patients with Diabetes Mellitus. Int. J. Low. Extrem. Wounds.

[22] Yousry A.H., Abdalhady A.M. (2010). Management of diabetic neuropathic ankle arthropathy by arthrodesis using an Ilizarov frame. Acta Orthop. Belg..

[23] Zarutsky E., Rush S.M., Schuberth J.M. (2005). The use of circular wire external fixation in the treatment of salvage ankle arthrodesis. J. Foot Ankle Surg..

[24] Pinzur M.S., Kelikian A. (1997). Charcot ankle fusion with a retrograde locked intramedullary nail. Foot Ankle Int..

[25] Pinzur M.S., Noonan T. (2005). Ankle Arthrodesis with a Retrograde Femoral Nail for Charcot Ankle Arthropathy. Foot Ankle Int..

[26] Dalla Paola L., Volpe A., Varotto D., Postorino A., Brocco E., Senesi A., Merico M., De Vido D., Da Ros R., Assaloni R. (2007). Use of a retrograde nail for ankle arthrodesis in Charcot neuroarthropathy: A limb salvage procedure. Foot Ankle Int..

[27] de Souza L.J. (2008). Charcot arthropathy and immobilization in a weight-bearing total contact cast. J. Bone Jt. Surg. Am. Vol..

[28] Caravaggi C.M., Sganzaroli A.B., Galenda P., Balaudo M., Gherardi P., Simonetti D., Ferraresi R., Farnetti A., Morandi A. (2012). Long-term Follow-up of Tibiocalcaneal Arthrodesis in Diabetic Patients with Early Chronic Charcot Osteoarthropathy. J. Foot Ankle Surg..

[29] Siebachmeyer M., Boddu K., Bilal A., Hester T.W., Hardwick T., Fox T.P., Edmonds M., Kavarthapu V. (2015). Outcome of one-stage correction of deformities of the ankle and hindfoot and fusion in Charcot neuroarthropathy using a retrograde intramedullary hindfoot arthrodesis nail. Bone Jt. J..

[30] Çinar M., Derincek A., Akpinar S. (2010). Tibiocalcaneal arthrodesis with posterior blade plate in diabetic neuroarthropthy. Foot Ankle Int..

[31] DeVries J.G., Berlet G.C., Hyer C.F. (2012). A Retrospective Comparative Analysis of Charcot Ankle Stabilization Using an Intramedullary Rod with or without Application of Circular External Fixator—Utilization of the Retrograde Arthrodesis Intramedullary Nail Database. J. Foot Ankle Surg..

[32] Shah N.S., De S.D. (2011). Comparative analysis of uniplanar external fixator and retrograde intramedullary nailing for ankle arthrodesis in diabetic Charcot’s neuroarthropathy. Indian J. Orthop..

[33] Myerson M.S., Alvarez R.G., Lam P.W.C. (2000). Tibiocalcaneal arthrodesis for the management of severe ankle and hindfoot deformities. Foot Ankle Int..

[34] Selby P., Young M., Boulton A. (1994). Bisphosphonates: A new treatment for diabetic Charcot neuroarthropathy?. Diabet. Med..

[35] Coukell A.J., Markham A. (1998). Pamidronate. Drugs Aging.

[36] Singer F.R., Minoofar P.N. (1995). Bisphosphonates in the treatment of disorders of mineral metabolism. Adv. Endocrinol. Metab..

